# Design of a Porous Cathode for Ultrahigh Performance of a Li-ion Battery: An Overlooked Pore Distribution

**DOI:** 10.1038/srep42521

**Published:** 2017-02-13

**Authors:** Jihwan Song, Junhyung Kim, Taewook Kang, Dongchoul Kim

**Affiliations:** 1 Department of Mechanical Engineering, Sogang University, Seoul 04107, Korea; 2 Department of Chemical and Biomolecular Engineering, Sogang University, Seoul 04107, Korea

## Abstract

Typical cathode materials of Li-ion battery suffer from a severe loss in specific capacity, and this problem is regarded as a major obstacle in the expansion of newer applications. To overcome this, porous cathodes are being extensively utilized. However, although it seems that the porosity in the cathode would be a panacea for high performance of LIBs, there is a blind point in the cathode consisting of porous structures, which makes the porous design to be a redundant. Here, we report the importance of designing the porosity of a cathode in obtaining ultrahigh performance with the porous design or a degraded performance even with increase of porosity. Numerical simulations show that the cathode with 40% porosity has 98% reduction in the loss of specific capacity when compared to the simple spherical cathode when the C-rate increases from 2.5 to 80 C. In addition, the loss over total cycles decreases from 30% to only about 1% for the cathode with 40% porosity under 40 C. Interestingly, however, the specific capacity could be decreased even with the increase in porosity unless the pores were evenly distributed in the cathode. The present analysis provides an important insight into the design of ultrahigh performance cathodes.

Typical cathode materials of Li-ion batteries (LIBs) suffer from a huge loss in specific capacity. Since this problem becomes increasingly severe as the C-rate and number of cycles increase when the batteries are operated (*i.e.*, low rate capability and low cycle stability)^1^,^2^, it is regarded as the main obstacle in the expansion of newer applications of such batteries, such as portable electronic devices, medical devices, hybrid electric vehicles (HEVs), and renewable energy storage.

Previous studies have revealed that the loss in specific capacity arises from the inherent material property, such as an unusually low diffusivity of Li-ion at the specific phase in the cathode material^3^,^4^. To overcome this inherently low diffusivity of Li-ion in the cathode, researchers have investigated ways with respect to the morphological design of the cathode, which can ensure improved insertion and extraction of Li-ion with a reduced diffusion pathway^3^,^4^,^5^,^6^,^7^,^8^,^9^,^10^,^11^,^12^,^13^,^14^,^15^. Initial efforts in this direction have focused on the design of one (1D) or two-dimensional (2D) cathode structures that provide a reduced diffusion pathway, for instance nanorods, nanowires, and nanosheets^16^,^17^,^18^,^19^,^20^,^21^,^22^. These studies have reported a considerably improved specific capacity, rate capability, and cycle stability in comparison with existing bulk structures. Although such structures have resulted in improved performances, they still have some problems. The low dimensional structures have trouble with effective interconnection as a cathode due to their low dimension, and this causes high electrical resistance and low mechanical stability^23^. In order to resolve this problem, three-dimensional (3D) structures that can provide the effect of a reduced diffusion pathway have been proposed. For example, a 3D porous structure or hollow structure has been suggested as the cathode structure. These structures have shown improved electrochemical performances as well as improved mechanical stability based on their effective interconnection^7^,^8^,^9^,^11^,^12^,^13^,^15^,^23^,^24^,^25^,^26^,^27^,^28^. Porous structures have been extensively utilized as cathodes to achieve improved performances because they can be relatively easily fabricated even in a 1D or 2D structure^29^,^30^,^31^,^32^,^33^. On the face of it, it seems that a porous cathode would be the best way to obtain high-performance LIBs. However, there is a blind point in the design of porous cathodes, which limits their usefulness.

Here, we report the importance of designing the porosity of a cathode such that the effect of porosity is amplified to achieve ultrahigh performance. The specific capacity and its loss are systematically evaluated with varying porosity, pore distribution, C-rate, and cycle number. Numerical simulations show that the specific capacity and its loss can be improved with increasing porosity of the cathode. Furthermore, the effect of porosity becomes significant as the C-rate and number of cycles increase. Interestingly, however, the porous cathode does not always guarantee an improved specific capacity with increased porosity. In other words, the specific capacity could decrease unless the pores are evenly distributed even though the porosity of the cathode increases.

## Results and Discussion

In order to evaluate the performance of a porous cathode, a 3D dynamic model based on previous studies related to the evolution of nano- and microstructures was developed^34^,^35^,^36^,^37^. Recently, a few studies based on phase field simulation have been reported, which describe the phase segregation of Li-ion battery electrodes^38^,^39^,^40^,^41^,^42^,^43^,^44^. However, importantly, the materials that they considered were limited to only two- or three-phase materials, namely FePO_4_ or Mn_2_O_4_. Furthermore, some of them have defined their total free energy with insufficient experimental evidence. Therefore, we employed a Fick’s law-based model to efficiently investigate our cathode that has the five phases rather than the construction of free energy for the phase field simulation that should include many unknown coefficients to describe the five different phases.

**Figure 1:**
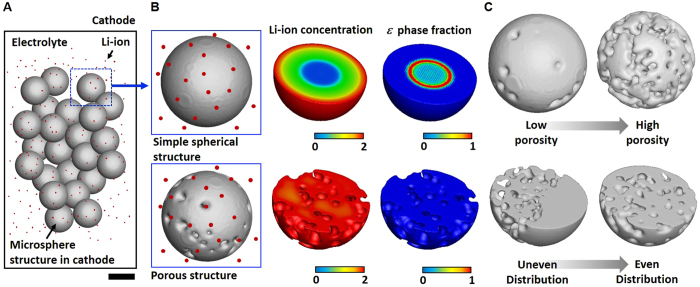
Design of the porous structure in the cathode for ultrahigh performance. (**A**) Illustration of the cathode in a Li-ion battery consisting of a microsphere structure, Li-ion, and an electrolyte. Scale bar: 500 nm. (**B**) Li-ion concentration and the *ε* phase fraction of a simple spherical structure and a porous structure in the cathode. (**C**) Design of a porous cathode with respect to porosity and the distribution of pores.

The schematic illustration of our cathode is shown in Fig. 1. The cathode comprises microsphere structures, Li-ion, and an electrolyte (Fig. 1A). During the operation of an LIB, the insertion and extraction of Li-ion occur in the microstructure of the cathode, and the performances of the LIB (*e.g.*, specific capacity, rate capability, and cycle stability) can be determined by the Li-ion concentration in the cathode. When Li-ions are inserted or extracted in the cathode, the phases of the cathode material are transformed depending on the concentration of the Li-ion. Since the diffusivity of the Li-ion in some phases could be considerably low, it would reduce the total amount of Li-ions in the cathode during the utilization of the cathode and would induce a loss in performance. For instance, as the concentration (*c*) of the Li-ion increases, the cathode made with vanadium pentoxide (V_2_O_5_), which is considered in this study, exhibits the following phase transformations: *α* (0.0 < *c* < 0.35), *ε* (0.1 < *c* < 1.0), *δ* (0.7 < *c* < 2.0)*, γ* (1.0 < *c* < 3.0), and *ω* (2.0 < *c* < 3.0)^1^ (see Supplementary Information Fig. S1). As shown in Fig. S1, each phase shows considerable difference in the diffusivity of the Li-ion for example, the *α, δ*, and *γ* phases have a value of about 10^−12^ cm^2^/s, while the *ε* and *ω* phases have values of about 10^−14^~10^−13^ cm^2^/s. Commonly, LIBs stop operating when the *ω* phase is generated to avoid further increase in the amount of the *ω* phase, whose formation is irreversible. Therefore, the diffusion of Li-ion into the cathode is interrupted by the formation of the *ε* phase during the discharging process owing to its significantly low diffusivity of Li-ion, which is about 10 times lower than that of the other phases (*i.e., α, δ*, and *γ*). The presence of porosity in the cathode could reduce the region of the cathode where Li-ion cannot reach due to the *ε* phase and result in full utilization of the cathode (Fig. 1B). As shown in Fig. 1C, the distribution of pores can be an important parameter in designing the microstructure of the cathode in addition to the porosity.

### Effect of porosity on specific capacity and rate capability of cathode

**Figure 2:**
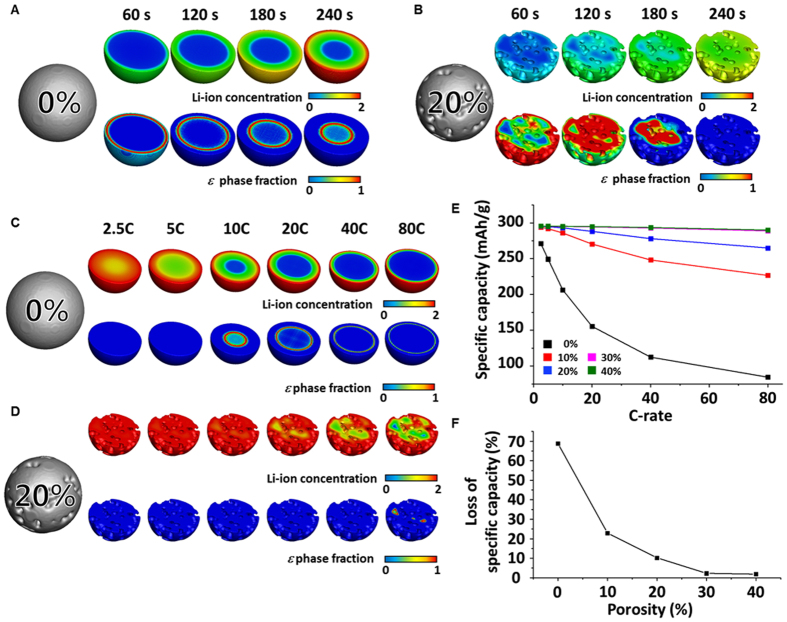
Ultrahigh specific capacity and rate capability of a cathode with porosity. Time-dependent image of Li-ion concentration and the *ε* phase fraction (**A**) in the simple spherical cathode and (**B**) in the cathode with 20% porosity under 10 C. Cross-sectional image of the Li-ion concentration and the *ε* phase fraction at the completion of discharge with increasing C-rate from 2.5 C to 80 C (**C**) in the simple spherical cathode and (**D**) in the cathode with 20% porosity. (**E**) Specific capacity with different porosities in the cathode according to different C-rates. (**F**) Loss in specific capacity with different porosities in the cathode as the C-rate increases from 2.5 C to 80 C.

First, we validated the feasibility of our model used to investigate the specific capacity of the cathode by comparison with the experimental results. As with experiments, the simple spherical and hollow spherical V_2_O_5_ cathodes were considered (see Supplementary Information Fig. S2 and Section 2.3 “Validation of model”). As shown by the inset (*i.e.*, blue box) in Fig. S2, at a cut-off potential of 2.2 V, the simple spherical and hollow spherical cathode show only 4.0% (*i.e.*, 8.7 mAh/g) and 0.6% (*i.e.*, 1.5 mAh/g) of difference with the experimental value, respectively. The specific capacities from the numerical model are in good agreement with the experimental observations^45^. To analyze the performance of the porous cathode, the difference in the porosity of the cathode is systematically investigated. The numerical simulations are performed using the developed 3D dynamic model (see Methods and Supplementary Information). Figure 2A shows the time-dependent Li-ion concentrations and the fraction of the *ε* phase during the discharging process for a simple spherical cathode with a porosity of 20%. The C-rate is set to be 10 C. In the simple spherical cathode, the Li-ion is accumulated outside of the *ε* phase because the diffusivity of the Li-ion shows an almost tenfold decrease when the phase is transformed from *α* to *ε* (see Supplementary Information Fig. S1). The *ε* phase remains until the discharging process is completed. As a result, the cathode has an unutilized part, which leads to the loss in specific capacity. On the other hand, since the 20% porosity provides a significantly increased surface area of the cathode and reduced diffusion pathway of the Li-ion into the cathode, the cathode with porosity is less affected by the low diffusivity of the *ε* phase. Indeed, the *ε* phase is transformed to the next phase (*i.e., δ* phase), which has about ten times higher diffusivity of the Li-ion, before the discharging process is finished and *ε* phase is not observed at the final stage (Fig. 2B). Thus, the porosity in the cathode allows its full utilization.

Additionally, the effect of porosity on the specific capacity was investigated with respect to the C-rates, by varying the C-rate from 2.5 to 80 (Fig. 2C and D). As shown in Fig. 2C, at low C-rates (*i.e.*, 2.5 C and 5 C), Li-ion can diffuse into the cathode even in the absence of porosity. Since Li-ions are slowly supplied to the cathode under low C-rates (*e.g.*, 2.5 C or 5 C), in comparison with high C-rates (*e.g.*, 10 C to 80 C), it allows the transformation of the *ε* phase to the next phase before the discharging process is finished (see Supplementary Information Fig. S2 to S7). As a result, even the simple spherical cathode can be fully utilized without the interruption of the *ε* phase on the diffusion of Li-ion into the cathode. When Li-ions are more rapidly supplied under a high C-rate such as 10 C, 20 C, 40 C, and 80 C, Li-ions are rapidly accumulated outside the *ε* phase. In this case, the *ε* phase remains until the discharge is finished and it results in the insufficient utilization of the cathode. In contrast, Fig. 2D shows that Li-ion can diffuse more effectively into the cathode with 20% porosity. Due to the significantly increased surface area and reduced pathway of diffusion, the impact of the *ε* phase on Li-ion diffusion is lower in the porous cathode when compared to the simple spherical cathode under the same C-rate. When the discharge is complete, the cathode with 20% porosity has the *ε* phase only at a C-rate of 80 C. As a result, the cathode with porosity can be fully utilized even under a high C-rate condition.

A quantitative analysis of the specific capacity with different cathode porosities is presented in Fig. 2E. The specific capacity mostly increases as the porosity in the cathode increases, and the effect of porosity becomes significant as the C-rate increases. The cathode with 40% porosity shows an improvement of 350% in the specific capacity over the simple spherical cathode at 80 C. Figure 2F shows the loss in specific capacity as the C-rate increases from 2.5 C to 80 C. Simulation results also show the high rate capability of the cathode with porosity. For example, the specific capacity of the simple spherical cathode decreases from 270 to 90 mAh/g as the C-rate increases from 2.5 to 80 C. On the other hand, that of the cathode with 40% porosity is only changed from 295 to 290 mAh/g for the same change in C-rate. The loss in specific capacity is dramatically reduced to be only about 5 mAh/g when the porosity of the cathode is 40%, while the simple spherical cathode exhibits a loss of about 180 mAh/g. The loss in specific capacity can be reduced by as much as 98% by introducing porosity in the cathode. Therefore, the problem of severe loss in specific capacity with increasing C-rate can be resolved by the design of porous cathodes.

### Effect of porosity on cycle stability of cathode

**Figure 3:**
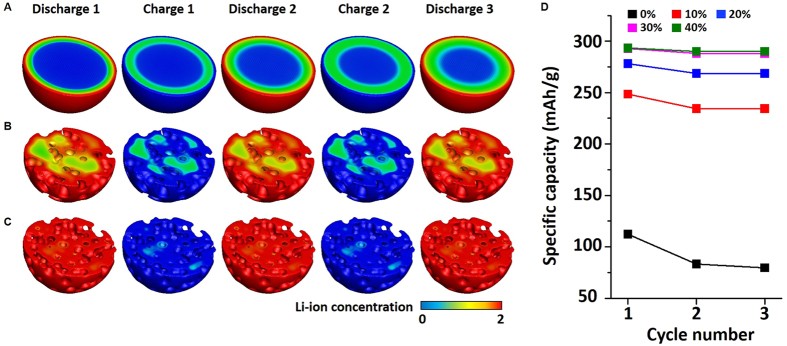
Ultrahigh cycle stability of the cathode with porosity. (**A**–**C**) Cross-sectional image of the Li-ion concentration at the completion of each discharging and charging process in the simple spherical cathode, cathode with 20% porosity, and cathode with 50% porosity, respectively. The C-rate is 40 C. (**D**) Specific capacity with different porosities of cathode during cycles.

Next, in order to investigate the cycle stability of the cathode, its specific capacity was studied with respect to the charging-discharging cycle (Fig. 3). Figure 3A to C represents the Li-ion concentration in the cathode over repeated charge and discharge cycles. The C-rate was set at 40 C, and the porosities of the cathode were 0%, 20%, and 40%. As shown in Fig. 3A and B, there is a considerable amount of Li-ions in the simple spherical cathode and the 20% porosity cathode even after the charging process is complete (*i.e.*, the region indicated in green after charging 1 and 2). In contrast, there is a relatively small amount of Li-ions in the 40% porosity cathode after the charging process (Fig. 3C). The cathode with 40% porosity provides easy transformation of the *ε* phase to the next phase due to its increased surface area and the reduced diffusion pathway. Li-ion can diffuse out of the cathode more easily than the others (*i.e.*, Fig. 3A and B) during the charging process.

The remaining Li-ions in the cathode after the charging process leads to a loss in specific capacity by reducing the amount of Li-ions that will be newly intercalated during the next discharge. The loss in specific capacity with cycle nuber is decreased by almost 90% as the porosity in the cathode increases; this is due to the increased surface area and the reduced diffusion pathway of the porous cathode. Quantitatively, the loss in specific capacity over all the cycles is only about 1% when the porosity of the cathode is 40%, while the simple spherical cathode shows about 30% loss in specific capacity (Fig. 3D).

### Effect of pore distribution on specific capacity of cathode

**Figure 4:**
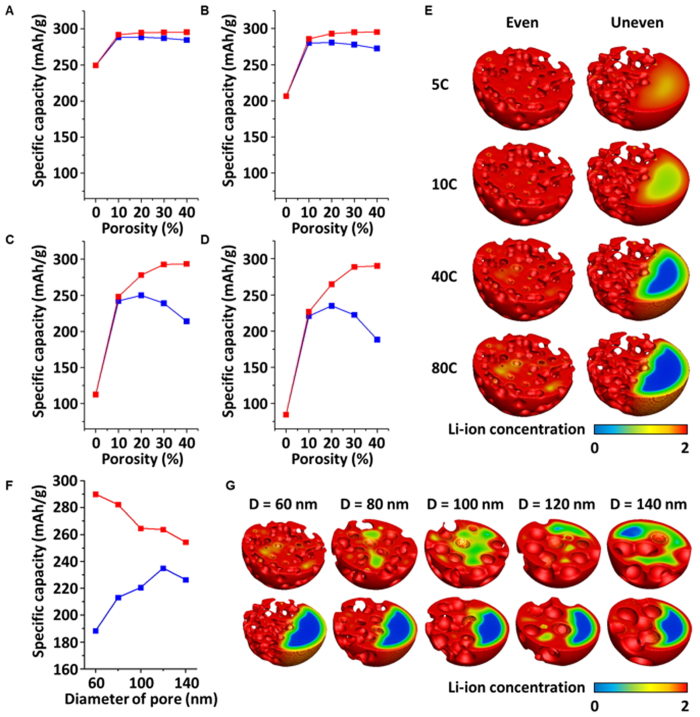
Effect of pore distribution on the specific capacity of the cathode. Specific capacities of the porous cathode with evenly distributed pores (red) and unevenly distributed pores (blue) at (**A**) 5 C, (**B**) 10 C, (**C**) 40 C, and (**D**) 80 C. (**E**) Cross-sectional image of the Li-ion concentration in the cathode with 40% porosity with an even distribution (left) and an uneven distribution (right). (**F**) Specific capacities of the cathode with evenly distributed pores (red) and unevenly distributed pores (blue) according to the diameter of the pore. The porosity is 40% and the C-rate is 80 C. (**G**) Cross-sectional image of the Li-ion concentration in the 40% porosity cathode according to the pore diameter variation: with even distribution (top) and uneven distribution of pores (bottom).

Next, the performance according to the distribution of pores in the cathode was investigated under 5, 10, 40, and 80 C-rate conditions. Porous cathodes that have an even and uneven distribution of pores were designed for each porosity. The specific capacities of each porous cathode with evenly distributed pores (*i.e.*, red line) and unevenly distributed pores (*i.e.*, blue line) are presented in Fig. 4A–D. Figure 4A to D presents the specific capacities at the 5, 10, 40, and 80 C-rates, respectively. The simulation reveals that the porous cathodes have up to 55% variation in the specific capacity according to the distribution of pores. Interestingly, the porosity of cathode could be a superfluous unless the pores are evenly distributed. Qualitatively, the cathode with well-distributed pores has a higher Li-ion concentration than the cathode with an uneven distribution of pores (Fig. 4E). This result can be explained by the pore-concentrated region that results in the restrictive use of cathode material. In other words, Li-ion is primarily intercalated at the pore-concentrated region due to the excessively shortened pathway of diffusion into the cathode and the much earlier onset of the *ε* phase than the other regions. Li-ions are increasingly accumulated in the region where the *ε* phase is generated due to the significantly lower diffusivity of Li-ion, and thus the discharging process is terminated without full utilization of the cathode. Additionally, the effect of the diameter of pores on the specific capacity was investigated (Fig. 4F and G). The porosity and C-rate were set at 40% and 80 C, respectively. Five different pore diameters, namely 60, 80, 100, 120, and 140 nm, were considered. As the diameter of the pore decreases, the specific capacity of the evenly designed porous cathode can be improved because the surface area increases with the decrease in pore diameter, while that of the unevenly designed porous cathode deteriorates (Fig. 4F). This deterioration originates from the pore-concentrated region and is due to the excessively shortened pathway of Li-ion diffusion as the pore diameter decreases, which results in early termination of the discharging process without full utilization of the cathode. The cathode with a pore diameter of 60 nm shows more restrictive utilization than that observed with a pore diameter of 120 or 140 nm (Fig. 4G), as the region where Li-ion cannot reach (*i.e.*, the blue region) for the cathode with 60 nm pores increases in comparison to that with 120 nm or 140 nm pores.

Consequently, designing porous cathodes by considering the distribution of pores may become important in achieving ultrahigh performance LIBs, as the distribution of pores determines whether the porous cathode will actually ensure ultrahigh performance or be redundant.

## Conclusion

In summary, we have reported the importance of designing the porosity of a cathode so as to obtain ultrahigh performance. A porous cathode can have a higher specific capacity than a simple spherical cathode, and the effect of porosity on the specific capacity becomes more prominent as the C-rate and number of cycles increase. The rate capability and the cycle stability are improved with increasing porosity in a cathode. As the C-rate increased from 2.5 C to 80 C, the loss in specific capacity was reduced to be only about 2% for the cathode with 40% porosity; in contrast, the simple spherical cathode showed about 70% loss in specific capacity. In addition, the loss in specific capacity over three cycles under 40 C was only about 1% when the porosity in the cathode was 40%, while the simple spherical cathode showed about 30% loss in specific capacity. Interestingly, the simulation revealed that the presence of porosity may become redundant unless the pores were evenly distributed. We expect that the analysis presented on the structural design of a cathode can provide an efficient way to achieve ultrahigh performance LIBs as an advanced power source.

## Methods

The diffusion flux of Li-ion into the cathode is represented by ***J***_*d*_ = −*D*∇*c*, where *D* and c denote the diffusivity and the concentration of Li-ion, respectively. Since we assume that V_2_O_5_ is a cathode material, the diffusivity of Li-ion in the cathode depends on the internal phases (*e.g., α, ε, δ, γ*, and *ω*) and the phases are determined by the Li-ion concentration in the cathode (see Supplementary Information Fig. S1)^46^. As shown in Fig. S1, the diffusivity of Li-ion in the cathode is not a constant and it is considered to be a function of the Li-ion. The phase and diffusivity of Li-ion at a given location in the cathode are determined according to the Li-ion concentration at that location, during the discharging and the charging processes. Further, when the Li-ion is inserted or is extracted through the cathode surface, it can be defined with the charging/discharging current density, *i*_*n*_ only at the surface boundary of cathode as ***J***_*i*_ · ***n*** = (*i*_*n*_/*F*)^47^,^48^, where ***n*** and *F* represent the surface normal vector and the Faraday constant.

Consequently, the total flux (***J*** = ***J***_*d*_ + ***J***_*i*_) combined with the conservation equation, 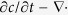 ***J*** = 0 gives the governing equation as follows:


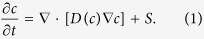


where *S* = ∇ · ***J***_*i*_. The value of 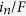 in the source term, 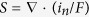
***n*** is determined with the given C-rate condition only at the surface boundary of cathode. (see Supplementary Information Section 2.1 “Model”). Then, the governing equation is normalized with the characteristic time *t*_c_, length *l*_c_, and diffusivity 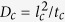 for computational convenience. The characteristic time and length are set to be *t*_c_ = 0.5 s and *l*_c_ = 5.0 nm, respectively. The normalized diffusivity, *D*_*n*_(*c*) = *D(c*)/*D*_*c*_, is defined as the characteristic diffusivity. Numerically, a semi-implicit Fourier spectral method is employed for high space resolution and fast computation^49^. In addition, the extrapolated gear (SBDF) scheme is combined for time integration^50^. In the Fourier space, the periodic boundary condition is assigned in all directions. Applying the scheme to Equation (1), the equation is effectively calculated with the Fourier transform, as follows:


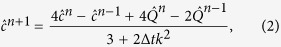



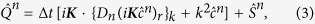


where the caret and subscript ***K*** stand for the Fourier transform. Subscript *r* denotes the inverse Fourier transform. The new concentration, 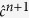 is obtained from the inverse Fourier transform. The specific capacity of the cathode is calculated with the average Li-ion concentration in the cathode, based on the theoretical specific capacity of Li_2_V_2_O_5_ (*i.e.*, 294.78 mAh/g)^51^. More details about the computational simulation can be found in the Supplementary Information.

The cathode is constructed from microspheres with a diameter of 500 nm. The porosity of the cathode is designed by controlling the distribution of spherical pores. Each spherical pore has a diameter of 60 nm and the porosity varies depending on the total number of pores in the cathode. The simulations are performed until the completion of discharge. The fully discharged state is recognized when the fraction of the *ω* phase in the cathode becomes 1% of the total volume of the cathode. This is because an LIB commonly stops operation when the *ω* phase is generated to avoid the increase in the fraction of the irreversible *ω* phase^51^. The fully charged state is assumed when the Li-ion concentration at the cathode surface becomes zero since the chemical reaction for the charging process is terminated when Li-ion do not exit at the cathode surface.

## Additional Information

**How to cite this article**: Song, J. *et al*. Design of a Porous Cathode for Ultrahigh Performance of a Li-ion Battery: An Overlooked Pore Distribution. *Sci. Rep.* **7**, 42521; doi: 10.1038/srep42521 (2017).

**Publisher's note:** Springer Nature remains neutral with regard to jurisdictional claims in published maps and institutional affiliations.

## Supplementary Material

Supplementary Information
