# Supplementary material for: Design of a Porous Cathode for Ultrahigh Performance of a Li-ion Battery: An Overlooked Pore Distribution
Source: Sci Rep. 2017 Feb 13;7:42521. doi: 10.1038/srep42521 (PMC5304199; doi:10.1038/srep42521)
Supplement: Supplementary Information [file srep42521-s1.pdf]

Supplementary Information

Design of a Porous Cathode for Ultrahigh Performance of a Li-ion Battery: An Overlooked Pore Distribution

Jihwan Song[1], Junhyung Kim[1], Taewook Kang[2], and Dongchoul Kim[1,*]

[1]Department of Mechanical Engineering, Sogang University, Seoul 04107, Korea
[2]Department of Chemical and Biomolecular Engineering, Sogang University, Seoul 04107, Korea.
*Corresponding Author: (Dongchoul Kim) Electronic mail: dckim@sogang.ac.kr

Table of Contents:

1. Supplementary Figures 1 to 8
2. Computational Details
   2.1 Model
   2.2 Numerical implementation
   2.3 Validation of model
3. Supporting References

Figure Legends

Fig. S1. The diffusivities of Li-ion in the V_2O_5 of cathode material according to the Li-ion concentration and the phase.

Fig. S2. Comparison between the numerical simulation (solid line) and the experimental (dashed line) performances. Specific capacity and voltage potential of the simple spherical (black line) and hollow spherical cathode (red line) at 0.2C. Both cathodes have 2 μm diameters, and the thickness of the hollow spherical cathode is 120 nm.

Fig. S3. Time-dependent image of Li-ion concentration and ε phase fraction in the simple spherical cathode and in the cathode with 10, 20, 30, and 40% porosity under 2.5C.

Fig. S4. Time-dependent image of Li-ion concentration and ε phase fraction in the simple spherical cathode and in the cathode with 10, 20, 30, and 40% porosity under 5C.

Fig. S5. Time-dependent image of Li-ion concentration and ε phase fraction in the simple spherical cathode and in the cathode with 10, 20, 30, and 40% porosity under 10C.

Fig. S6. Time-dependent image of Li-ion concentration and ε phase fraction in the simple spherical cathode and in the cathode with 10, 20, 30, and 40% porosity under 20C.

Fig. S7. Time-dependent image of Li-ion concentration and ε phase fraction in the simple spherical cathode and in the cathode with 10, 20, 30, and 40% porosity under 40C.

Fig. S8. Time-dependent image of Li-ion concentration and ε phase fraction in the simple spherical cathode and in the cathode with 10, 20, 30, and 40% porosity under 80C.

1. Supplementary Figures

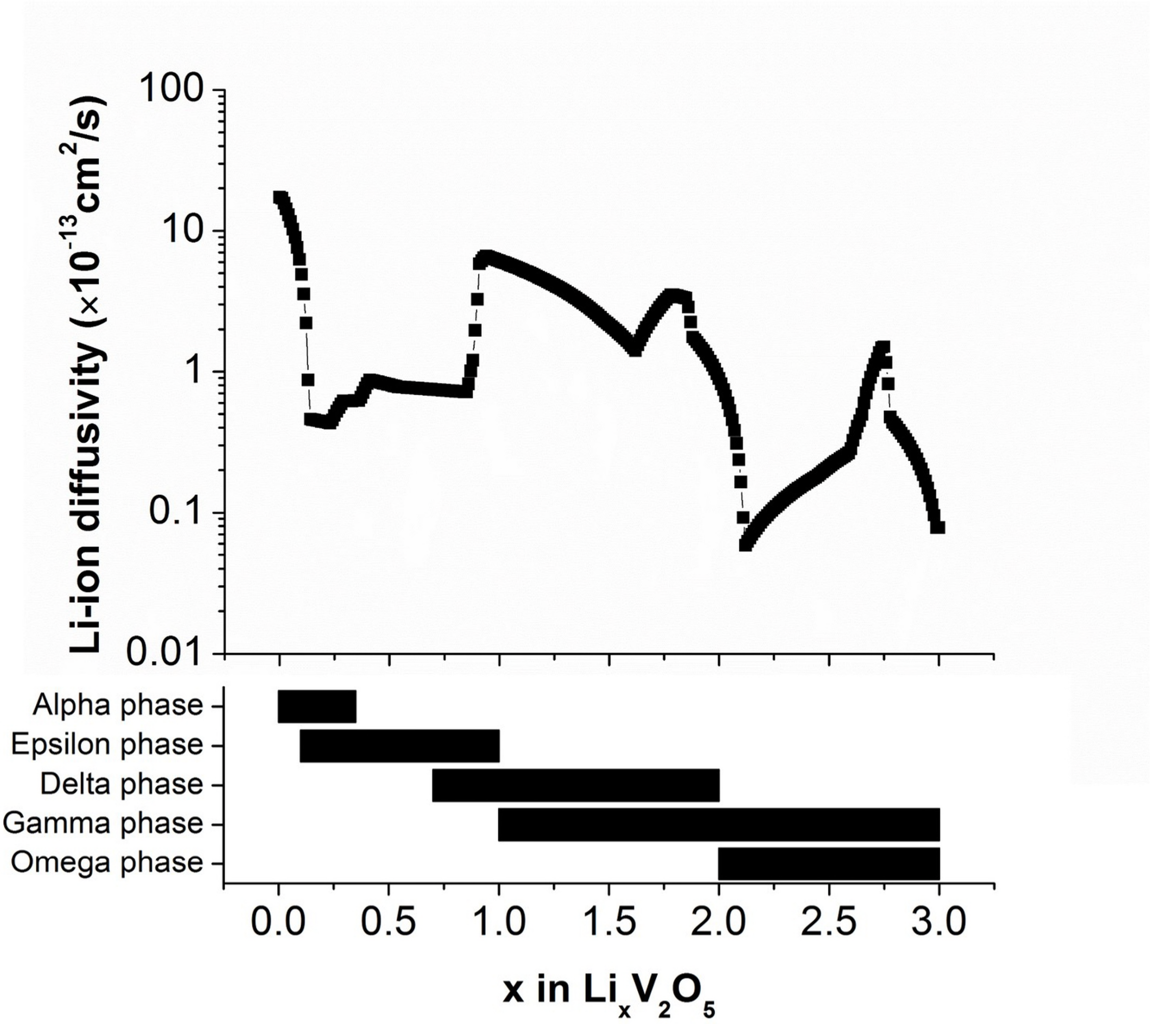

Figure S1. The diffusivities of Li-ion in the V_2O_5 of cathode material according to the Li-ion concentration and the phase.

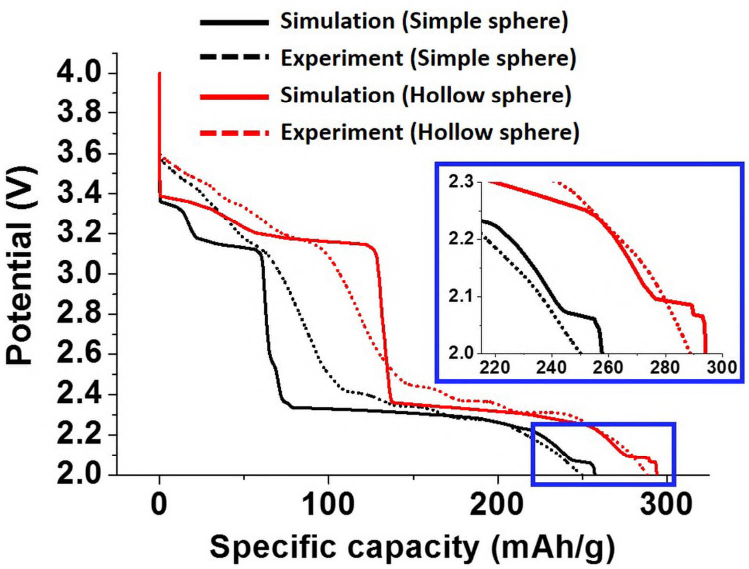

Figure S2. Comparison between the numerical simulation (solid line) and the experimental (dashed line) performances. Specific capacity and voltage potential of the simple spherical (black line) and hollow spherical cathode (red line) at 0.2C. Both cathodes have 2 μm diameters, and the thickness of the hollow spherical cathode is 120 nm.

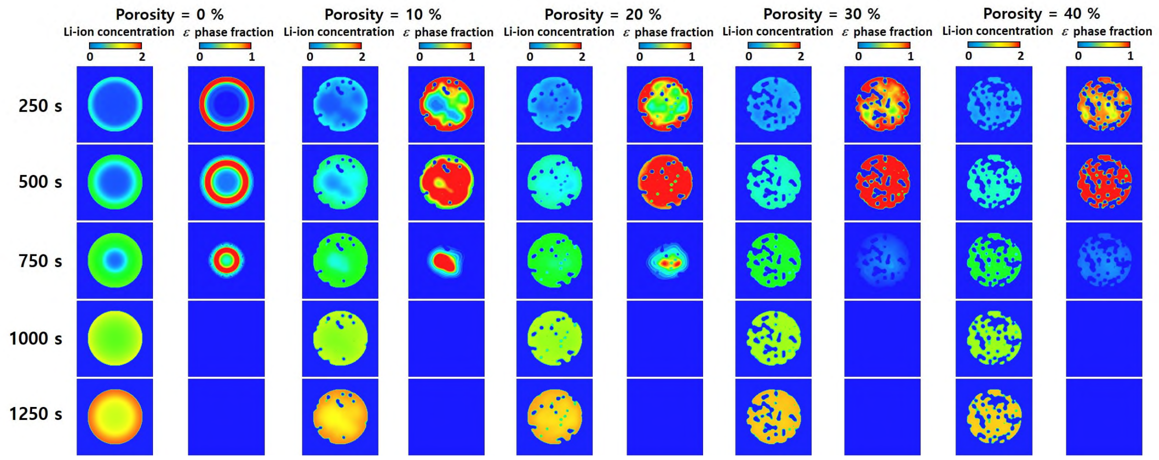

Figure S3. Time-dependent image of Li-ion concentration and ε phase fraction in the simple spherical cathode and in the cathode with 10, 20, 30, and 40% porosity under 2.5C.

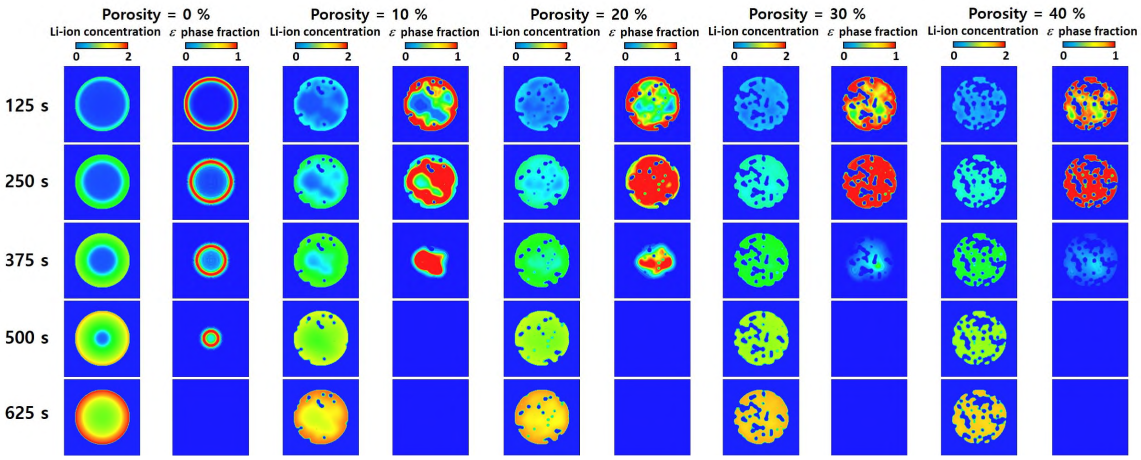

Figure S4. Time-dependent image of Li-ion concentration and ε phase fraction in the simple spherical cathode and in the cathode with 10, 20, 30, and 40% porosity under 5C.

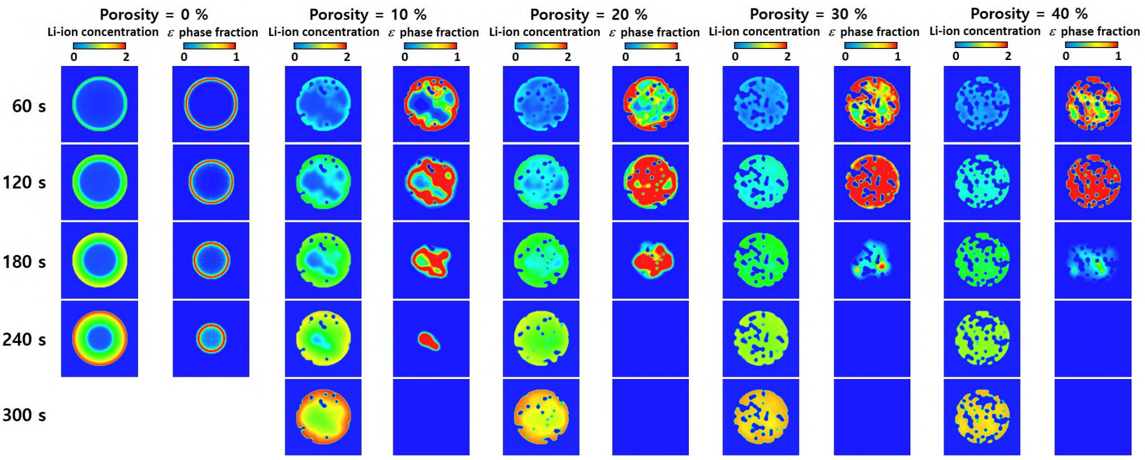

Figure S5. Time-dependent image of Li-ion concentration and ε phase fraction in the simple spherical cathode and in the cathode with 10, 20, 30, and 40% porosity under 10C.

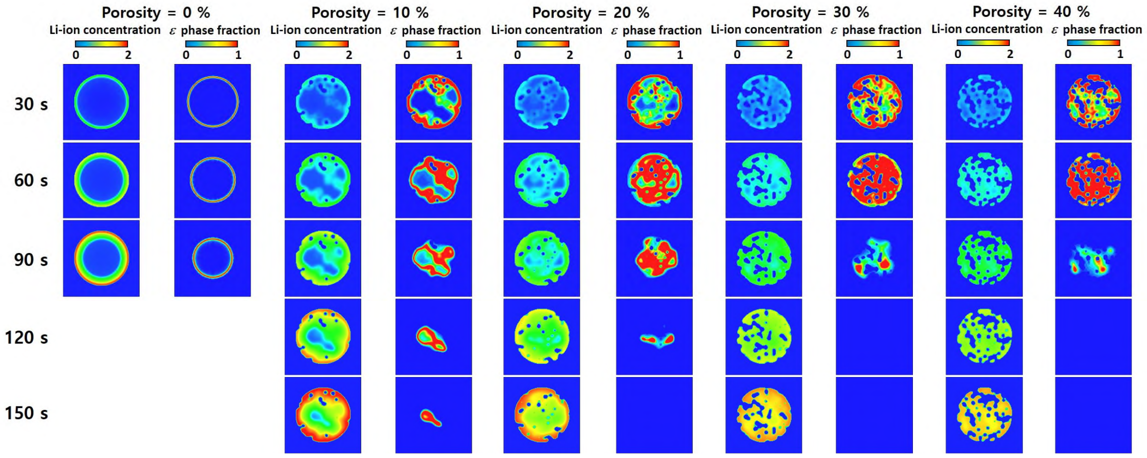

Figure S6. Time-dependent image of Li-ion concentration and ε phase fraction in the simple spherical cathode and in the cathode with 10, 20, 30, and 40% porosity under 20C.

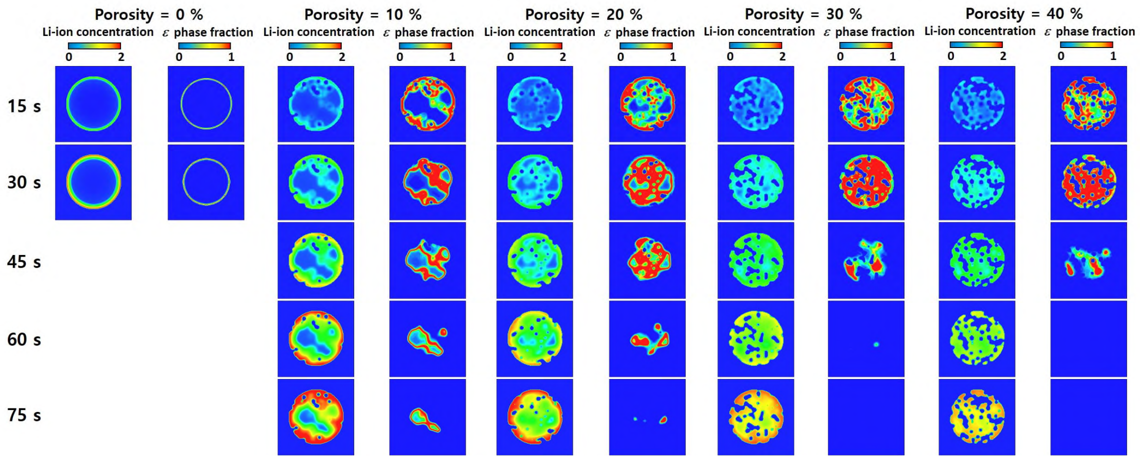

Figure S7. Time-dependent image of Li-ion concentration and ε phase fraction in the simple sphere-shaped cathode and in the cathode with 10, 20, 30, 40% porosity under 40C.

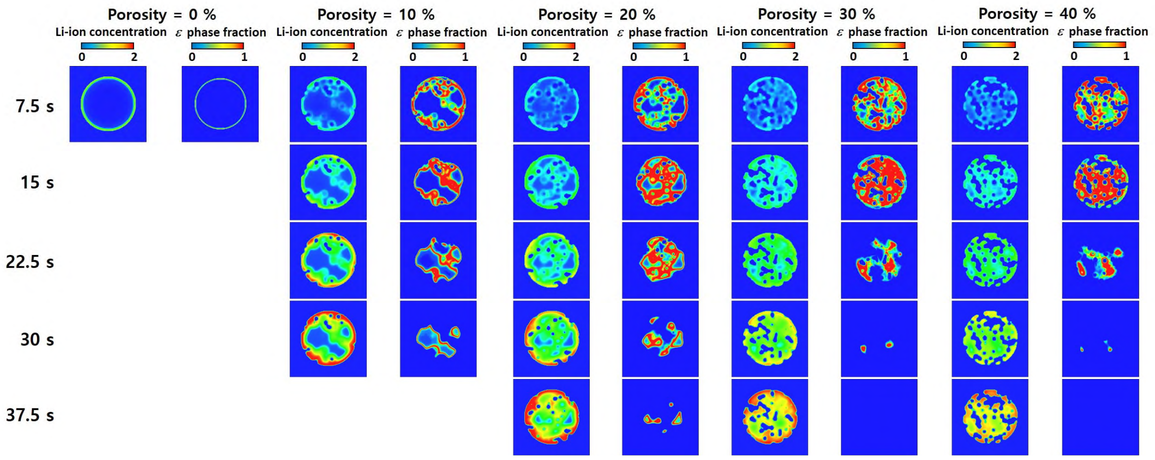

Figure S8. Time-dependent image of Li-ion concentration and ε phase fraction in the simple sphere-shaped cathode and in the cathode with 10, 20, 30, 40% porosity under 80C.

2. Computational Details

2.1 Model

The governing equation with the flux and the source of Li-ion is as follows:

$$\frac{\partial c}{\partial t} = \nabla \cdot \left[D(c) \nabla c \right] + S , \tag{1}$$

where source term, S is given by $\nabla \cdot (i_n / F)\boldsymbol{n}$. In the source term, the value of i_n / F is set according to the C-rate condition as an input data. The current density, i_n is assume to be constant during the charging/discharging process with the galvanostatic condition[1]. For example, during the discharging process, the total amount of Li-ion per time inserted through the surface boundary of cathode can be described by $\int_V \nabla \cdot (i_n / F)(-\boldsymbol{n}) dV$. On the other hand, if the C-rate is set, the increased amount of Li-ion per time in the cathode can be easily obtained as c_{max} × (C-rate/3600 s) × V, based on the definition of C-rate[2]. V and c_{max} are the cathode volume and the concentration when the cathode is fully charged, respectively. Because the total amount of Li-ion per time inserted through the surface boundary should be equal to the increased amount of Li-ion per time in the cathode, it gives $\int_V \nabla \cdot (i_n / F)(-\boldsymbol{n}) dV$ = c_{max}× (C-rate/3600 s) × V. Thus, i_n / F is obtained by c_{max}× (C-rate/3600 s) × $V / \int_V \nabla \cdot (-\boldsymbol{n}) dV$. According to the C-rate condition, different value of i_n / F is set at the surface boundary as an input data.

2.2 Numerical implementation

In the three-dimensional simulation, it is particularly important that the approach ensure efficiency and stability for the time integration in three-dimensional simulations. In addition, a numerical approach that ensures a high spatial resolution is required to resolve the high-order derivatives in the governing equation.

To achieve both high space resolution and fast computation, we propose an efficient semi-implicit Fourier spectral method. Numerically, the linear term is implicitly treated and the nonlinear term is explicitly treated in the semi-implicit method in order to allow for larger time steps without losing numerical stability[3]. Additionally, the Extrapolated Gear (SBDF) scheme is also combined to achieve stability for the time integration. This Extrapolated Gear (SBDF) scheme can provide the strongest high-modal decay among the second-order multistep methods[4]. The required damping for the very high frequencies in the diffusion equation is satisfied without a harsh time-step constraint. The periodic boundary condition is assigned along the x, y, and z directions in the Fourier space, and thus, the effect of the surrounding

boundary is ignored. Applying the scheme to the governing equation, we obtain the following discretized form:

$$\frac{3}{2}c^{n+1} - 2c^n + \frac{1}{2}c^{n-1} = \Delta t(\nabla^2 c^{n+1}) + 2Q^n - Q^{n+1}, \tag{2}$$

$$Q^n = \Delta t\left[\nabla \cdot \left(D\nabla c^n\right) - \nabla^2 c^n + \boldsymbol{S}^n\right]. \tag{3}$$

The equations (2) and (3) are effectively calculated with the Fourier transform:

$$\hat{c}^{n+1} = \frac{4\hat{c}^n - \hat{c}^{n-1} + 4\hat{Q}^n - 2\hat{Q}^{n-1}}{3 + 2\Delta t k^2}, \tag{4}$$

$$\hat{Q}^n = \Delta t\left[\boldsymbol{iK} \cdot \left\{D_n\left(\boldsymbol{iK}\hat{c}^n\right)_r\right\}_k + k^2\hat{c}^n\right] + \hat{\boldsymbol{S}}^n, \tag{5}$$

where the caret and subscript $\boldsymbol{K}$ stand for the Fourier transform. Vector $\boldsymbol{K}$ denotes the wave vector in Fourier space, and $k^2 = k_1^2 + k_2^2 + k_3^2$. The subscript r denotes the inverse Fourier transform. The new concentration c^{n+1} is obtained from $\hat{c}^{n+1}$ by the inverse Fourier transform. The procedure is repeated until a prescribed time.

2.3 Validation of model

We have validated the feasibility of our model for the investigation of the cathode specific capacity. We found that the specific capacity determined using our model is significantly accurate in comparison with the experimentally determined cathode specific capacity[5]. The simulations performed with the simple spherical cathode and the hollow spherical cathode as same with the structures of experiment. The cathode materials used was vanadium pentoxide (V_2O_5). The diameters of both cathodes were set to be 2 μm, and the thickness of hollow spherical cathode was 120 nm because the cathode diameters were in the range from 1.5 μm to 2 μm and the thickness of the hollow spherical cathode was in the range from 100 nm to 200 nm in the experiment.

In the simulation, the specific capacity was obtained with the average Li-ion concentration in the cathode, based on the theoretical specific capacity of $Li_2V_2O_5$[6]. For example, when the average Li-ion concentration in the cathode is 2.0, the specific capacity of the cathode corresponds to 294.78 mAh/g. Further, the voltage potential ϕ is obtained using the Butler-Volmer equation and the current density at the

surface i_{surf} is obtained as $\phi = \left(RT / \alpha F\right)\ln\left[\left(i_{surf} / i_0 + \sqrt{i_{surf} / i_0 + 4}\right) / 2\right] + U_{OCP}$ [7]. *R*, *T*, *α*, *F*, and i_0 are the gas constant, temperature, charge-transfer coefficient, Faraday constant, and exchange current density, respectively, and these are set to be 8.314 J/mol·K, 300 K, 0.5 [8], 9.65×10^4 J/*V*·mol, 1.6×10^{-3} mA/cm^2 [9]. The open circuit voltage, U_{OCP} is fitted from the experimental data[10].

Figure S2 shows the simulation results for the specific capacity and the voltage potential. As shown in the inset (*i.e.*, blue box) of Figure S2, the simulation results are significantly coincident with the experimentally determined specific capacity at the cut-off potential. For instance, at the 2.2 V cut-off potential, the numerical specific capacity of the simple spherical cathode (*i.e.*, black solid line) was 226.4 mAh/g, while the experimental specific capacity was 217.7 mAh/g (*i.e.*, black dashed line). They showed only 4.0% difference (*i.e.*, 8.7 mAh/g). Even if the cut-off potential was changed to be 2.1 V or 2.0 V, they still showed only 1.6% and 3.0% difference, respectively. In case of the hollow spherical cathode, at the 2.2 V cut-off potential, the numerical specific capacity was 263.2 mAh/g (*i.e.*, red solid line), while the experimental specific capacity was 264.7 mAh/g (*i.e.*, red dashed line). At 2.1 and 2.0 V cut-off potential, only 0.7% and 1.9% difference is observed. Because the hollow spherical structure has a reduced diffusion pathway, both numerical and experimental specific capacity values become almost comparable to the theoretical specific capacity (*i.e.*, 294.78 mAh/g)[6]. Thus, from the simulation results, we confirmed that the specific capacity determined using our model is highly consistent with the experimental observations because they showed an error within only 5%.

3. Supporting References

1 Lim, C., Yan, B., Yin, L. L. & Zhu, L. K. Simulation of diffusion-induced stress using reconstructed electrodes particle structures generated by micro/nano-CT. *Electrochim. Acta* **75**, 279-287 (2012).

2 He, G., Ji, X. L. & Nazar, L. High "C" rate Li-S cathodes: Sulfur imbibed bimodal porous carbons. *Energ. Environ. Sci.* **4**, 2878-2883 (2011).

3 Chen, L. Q. & Shen, J. Applications of semi-implicit fourier-spectral method to phase field equations. *Comput. Phys. Commun.* **108**, 147-158 (1998).

4 Ethier, M. & Bourgault, Y. Semi-implicit time-discretization schemes for the bidomain model. *Siam J. Numer. Anal.* **46**, 2443-2468 (2008).

5 Liu, J., Zhou, Y. C., Wang, J. B., Pan, Y. & Xue, D. F. Template-free solvothermal synthesis of yolk-shell V_2O_5 microspheres as cathode materials for Li-ion batteries. *Chem. Commun.* **47**, 10380-10382 (2011).

6 Afyon, S., Krumeich, F., Mensing, C., Borgschulte, A. & Nesper, R. New high capacity cathode materials for rechargeable Li-ion batteries: Vanadate-borate glasses. *Sci. Rep.-UK* **4**, 7113 (2014).

7 Li, X. Y., Xiao, M., Choe, S. Y. & Joe, W. T. Modeling and analysis of $LiFePO_4$/carbon battery considering two-phase transition during galvanostatic charging/discharging. *Electrochim. Acta* **155**, 447-457 (2015).

8 Smekens, J. *et al.* A modified multiphysics model for lithium-ion batteries with a $Li_xNi_{1/3}Mn_{1/3}Co_{1/3}O_2$ electrode. *Electrochim. Acta* **174**, 615-624 (2015).

9 Sequeira, C. A. C. & Santos, D. M. F. Tungsten oxide electrochromic windows with lithium polymer electrolytes. *J. Electrochem. Soc.* **157**, J202-J207 (2010).

10 McGraw, J. M. *et al.* Li-ion diffusion measurements in V_2O_5 and $Li(Co_{1-x}Al_x)O_2$ thin-film battery cathodes. *Electrochim. Acta* **45**, 187-196 (1999).
